# A Novel Algicidal Bacterium and Its Effects against the Toxic Dinoflagellate *Karenia mikimotoi* (Dinophyceae)

**DOI:** 10.1128/spectrum.00429-22

**Published:** 2022-05-26

**Authors:** Xinguo Shi, Yazhen Zou, Wenhuang Zheng, Lemian Liu, Youping Xie, Ruijuan Ma, Jianfeng Chen

**Affiliations:** a Technical Innovation Service Platform for High Value and High Quality Utilization of Marine Organism, Fuzhou University, Fuzhou, China; b Fujian Engineering and Technology Research Center for Comprehensive Utilization of Marine Products Waste, Fuzhou University, Fuzhou, China; c Fuzhou Industrial Technology Innovation Center for High Value Utilization of Marine Products, Fuzhou University, Fuzhou, China; Technical University of Denmark

**Keywords:** *Karenia mikimotoi*, algicidal bacterium, dinoflagellate, harmful algal bloom

## Abstract

The toxic dinoflagellate *Karenia mikimotoi* is a harmful algal bloom-forming species in coastal areas around the world. It produces ichthyotoxins and hemolytic toxins, with deleterious effects on marine ecosystems. In this study, the bacterium *Pseudoalteromonas* sp. FDHY-MZ2, with high algicidal efficiency against *K. mikimotoi*, was isolated from a bloom event. Based on the results, it completely lysed *K. mikimotoi* cells within 24 h 0.5% (vol/vol), with the algicidal activity of the supernatant of the bacterium culture. Algal cell wall fragmentation occurred, leading to cell death. There was a marked decline in various photochemical traits. When treated with the supernatant, cellulase, pheophorbide a oxygenase (PAO) and cyclin B genes were significantly increased, suggesting induced cell wall deterioration, chloroplast degradation and cell cycle regulation of *K. mikimotoi* cells. In addition, the expression levels of reactive oxygen species (ROS) scavenging gene was significantly inhibited, indicating that the ROS removal system was damaged. The bacterial culture was dried to obtain the spray-dried powder, which showed algicidal activity rates of 92.2 and 100% against a laboratory *K. mikimotoi* culture and a field microcosm of *Karlodinium* sp. bloom within 24 h with the addition of 0.04% mass fraction powder. Our results demonstrate that FDHY-MZ2 is a suitable strain for *K. mikimotoi* and *Karlodinium* sp. blooms management. In addition, this study provides a new strategy for the anthropogenic control of harmful algal bloom-forming species *in situ*.

**IMPORTANCE** *K. mikimotoi* is a noxious algal bloom-forming species that cause damaging of the aquaculture industry and great financial losses. Bacterium with algicidal activity is an ideal agency to inhibit the growth of harmful algae. In this approach application, the bacterium with high algicidal activity is required and the final management material is ideal for easy-to-use. The algicidal characteristics are also needed to understand the effects of the bacterium for managing strategy exploration. In this study, we isolated a novel algicidal bacterium with extremely high lysis efficiency for *K. mikimotoi*. The algicidal characteristics of the bacterium as well as the chemical and molecular response of *K. mikimotoi* with the strain challenge were examined. Finally, the algicidal powder was explored for application. The results demonstrate that FDHY-MZ2 is suitable for *K. mikimotoi* and *Karlodinium* sp. blooms controlling, and this study provides a new strategy for algicidal bacterium application.

## INTRODUCTION

Globally, harmful algal blooms (HABs) have occurred frequently in coastal areas in recent decades and negatively impact fishery resources, public health and marine ecosystems (1). According to recent studies, some heterotrophic bacterial lineages have significant effects on algal blooms due to their promotion or inhibition of the growth of bloom-forming species (2–4). Some specific bacteria, such as Pseudoalteromonadaceae and Alteromonadaceae, are associated with the termination of algal blooms (5) and play potential roles in HAB management (6–10).

Most of the algicidal bacteria are Gram-negative bacteria. Of these, over 50% belong to the Cytophaga-Flavobacteria-Bacteroidetes (CFB) group, and the remaining ones are Proteobacteria (10). A small number of algicidal bacteria are Gram-positive bacteria, namely, Firmicutes and Actinobacteria (10, 11). Various algicidal bacteria show a broad activity on microalgae from different phyla (9, 12). For instance, the CFB-bacterial strains S03 and 41-DBG2 are active against both dinoflagellate lineages, including *Karenia* spp. and *Alexandrium* spp. and diatom species, including *Pseudo-nitzschia multiseries* and Skeletonema costatum (12). Some algicidal bacteria can inhibit or lyse specific groups or certain species. Hare et al. isolated *Shewanella* IRI-160 strain (13), belonging to Gammaproteobacteria, which showed strong algicidal activity against dinoflagellates, whereas a diatom, a cryptophyte and a chlorophyte were not affected (14). Some algicidal bacteria belonging to the same genus can affect different microalgae species. The genus *Alteromonas* is active against various phytoplankton (10, 11). In a recent study, several *Alteromonas* strains were isolated, and all showed algicidal activity against *Karenia mikimotoi* (15). In contrast, strain FDHY-03, from the *Alteromonas* genus, had no algicidal effects on *K. mikimotoi* (9), suggesting that lysis activity is species-specific of this bacterium.

When challenged by algicidal bacteria, the target cell shows various physiological responses and morphological changes, resulting in cell death (16–19). These responses include cellular photosynthetic pigment degradation (17), reactive oxygen species (ROS) generation (20) and decreased photosynthetic electron transport rate (16). In addition, cell cycle arrest and programmed cell death (PCD) are observed in three dinoflagellate species, including *Prorocentrum minimum*, Karlodinium veneficum and *Gyrodinium instriatum* treated with algicidal bacteria (21). However, at the genetic level, the mechanisms underlying these responses during cell death are still unclear.

The dinoflagellate *K. mikimotoi* is a noxious algal bloom-forming species that causes mass mortalities in the halobios worldwide, significantly damaging the aquaculture industry (22, 23). It frequently booms in China, which causes great financial losses. In 2012, a single bloom event of this species in the Fujian Province of China led to an economic loss of more than 2 billion Yuan (22). In that event, the bloom caused massive mortality of abalone, *Haliotis discus hannai*. *Karlodinium* is a sister genus of *Karenia*, both of them belong to the family Kareniaceae, with similar cell structures and ecological characteristics. They were reported to bloom simultaneously in cases (24–26). In this study, a new algicidal bacterial strain, FDHY-MZ2, with high lysis efficiency for *K. mikimotoi*, was isolated and identified from surface seawater at the decline phase of a *K. mikimotoi* bloom. To understand the underlying mechanisms of the activity of the FDHY-MZ2 strain against *K. mikimotoi*, the basic characteristics and algicidal action mode of this strain were investigated. In addition, the morphological profile of *K. mikimotoi* during cell death was investigated microscopically. To illustrate the molecular mechanisms of the response of *K. mikimotoi* to FDHY-MZ2, the expression profiles of genes related to ROS generation, chloroplast synthesis and degradation, cell wall formation and deterioration and cell cycle regulation of the algae were observed. To exploit this bacterium for the management of *K. mikimotoi*, the bacterial culture was used to produce algicidal powder by spray drying. Subsequently, the activity of the spray-dried powder was tested against a laboratory *K. mikimotoi* culture and field microcosms of *Karlodinium* sp.-dominated blooms.

## RESULTS

### Effects of strain FDHY-MZ2 on *K. mikimotoi*.

**FIG 1:**
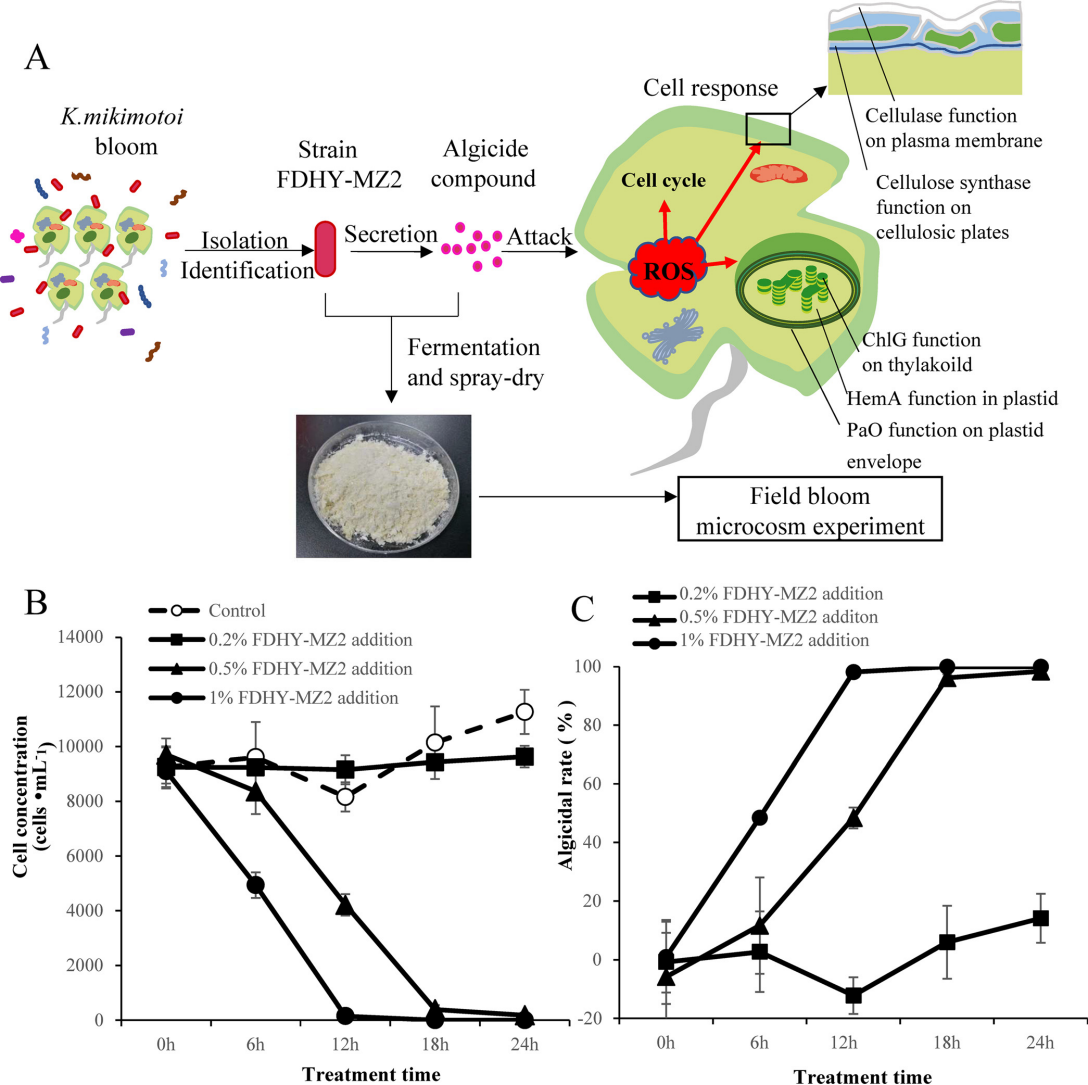
General diagram of experiment design (A) and algicidal activity of strain FDHY-MZ2 against *K. mikimotoi* (B-C). Growth dynamics (B) and algicidal rate (C) of algal cultures with different volumes. Error bars indicate ± standard deviation of biological triplicates.

The bacterium FDHY-MZ2, with high algicidal efficiency against K. mikimotoi, was isolated from the surface seawater of a bloom event (Fig. 1A). In the primary screening of alga-lysing bacteria, no *K. mikimotoi* cell was observed after 24 h treatment with 2% (vol/vol) FDHY-MZ2. In both 0.5 and 1% treatment groups, algal cells were completely lysed after treatment for 24 h (Fig. 1B). Compared to the axenic-treating culture with sterile 2216E (control), the algicidal rate reached 98.17% at 12 h in the 1% treatment groups. In the 0.5% treatment groups, the algicidal rate reached 96.18% at 18 h (Fig. 1C). However, no obvious algicidal activity was observed in the 0.2% treatment group (Fig. 1B–C). The 2% vol/vol data were not shown due to 100% algicidal rate of this volume.

**FIG 2:**
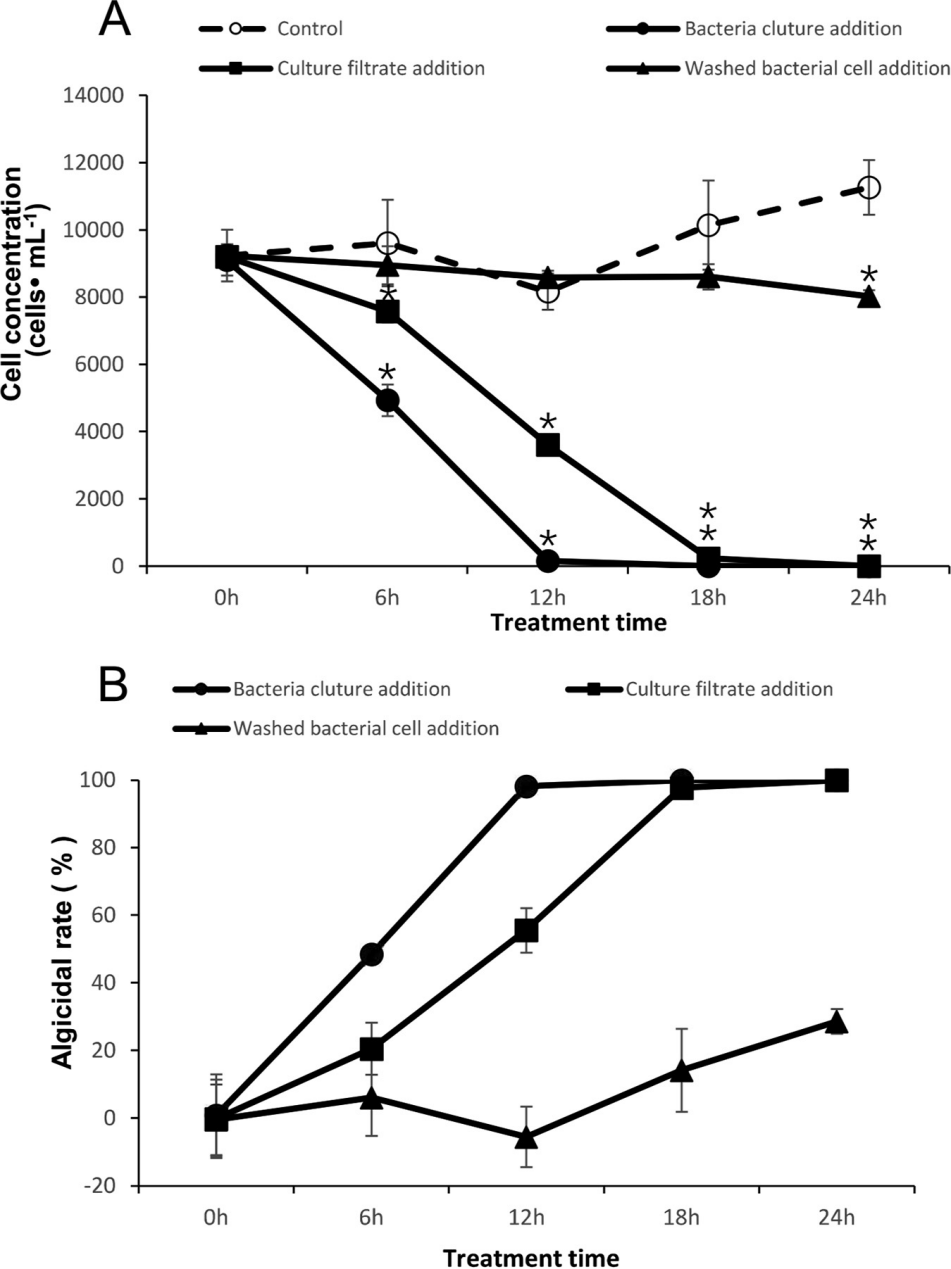
Algicidal modes of strain FDHY-MZ2 against *K. mikimotoi*. Growth dynamics (A) and algicidal rate (B) of algal cultures with different fractions of bacterial culture. Error bars indicate ± standard deviation of biological triplicates. For A, Statistical analyses were performed between cell density of each fraction treated algal culture and control culture. Black * represents statistical significance.

In the algicidal mode of action experiment, both algal cultures with the addition of supernatant and the bacterial strain showed high algicidal activity over 24 h (Fig. 2A and B). Compared to control group, the cell concentration decreased significantly in both supernatant and the bacterial strain addition cultures on 6 h, 12 h, 18 h and 24 h time point. No significant decrease was detected in washed bacterial cells addition cultures on 6 h 12 h and 18 h time point. On 24 h time point, cell concentration of this culture decreased 14.49% with significant.

**FIG 3:**
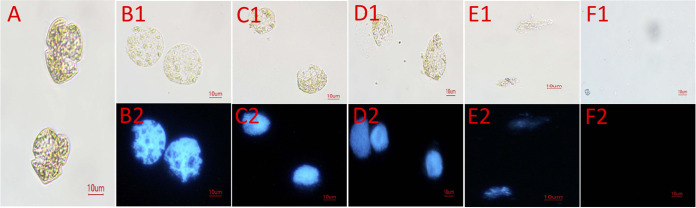
Changes in the morphology and nuclei of *K. mikimotoi* cells with FDHY-MZ2 challenging. A, bright-field illumination of *K. mikimotoi* cells; B1-F1 indicate algal cells treated with FDHY-MZ2 for 0, 6, 12, 18 and 24 h under bright-field, respectively. B2-F2 represents UV illumination of DAPI (5 g/mL)-stained nuclei for the cells B1-F1, respectively.

Algal lysis by strain FDHY-MZ2 was observed on *K. mikimotoi* microscopically (Fig. 3). Cell lysis started with the partial breaking of the cell membrane, followed by crackling of the whole cell and swelling. Finally, the cells burst and released cellular compounds. Based on DAPI staining results, the nucleus and proteins were damaged, along with cell wall/membrane degradation.

### Identification of strain FDHY-MZ2 and its lytic effects on various HAB species.

**FIG 4:**
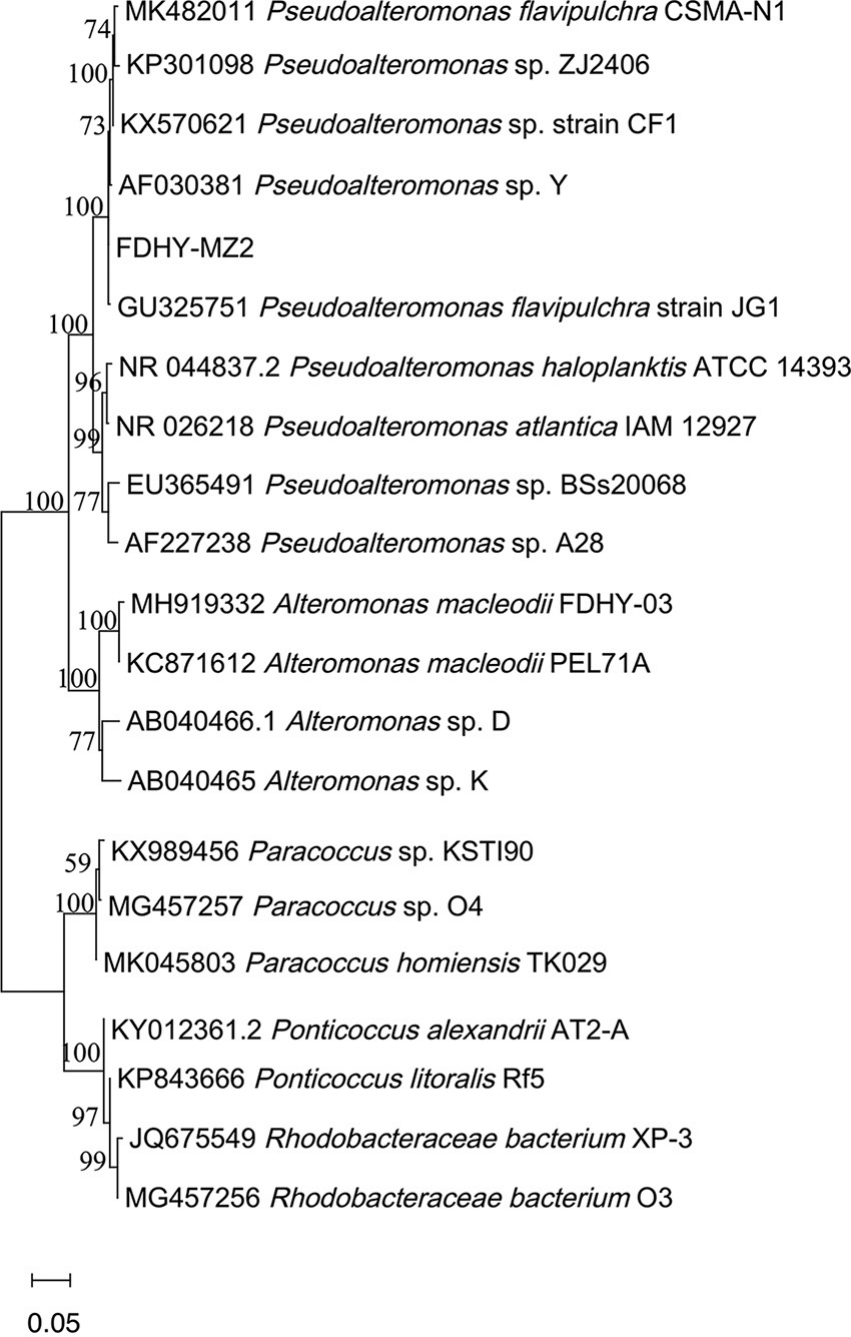
Phylogenetic tree of known algicidal bacteria, including strain FDHY-MZ2. Tree topology shown is obtained from the neighbor-joining analysis. Support of node > 50% is shown. The codes in front of the names are the GenBank accession numbers.

Based on the 16s rDNA sequencing result, strain FDHY-MZ2 was identified as a species of the genus *Pseudoalteromonas* (Fig. 4). The sequence of this strain (GenBank accession number: OM060674) exhibited the highest similarity (99.73%) to that of Pseudoalteromonas flavipulchra strain JG1, with the shortest genetic distance among the Pseudoalteromonadaceae species and the related species (Fig. 4). We used *Pseudoalteromonas* sp. FDHY-MZ2 as a reference strain in this study.

**FIG 5:**
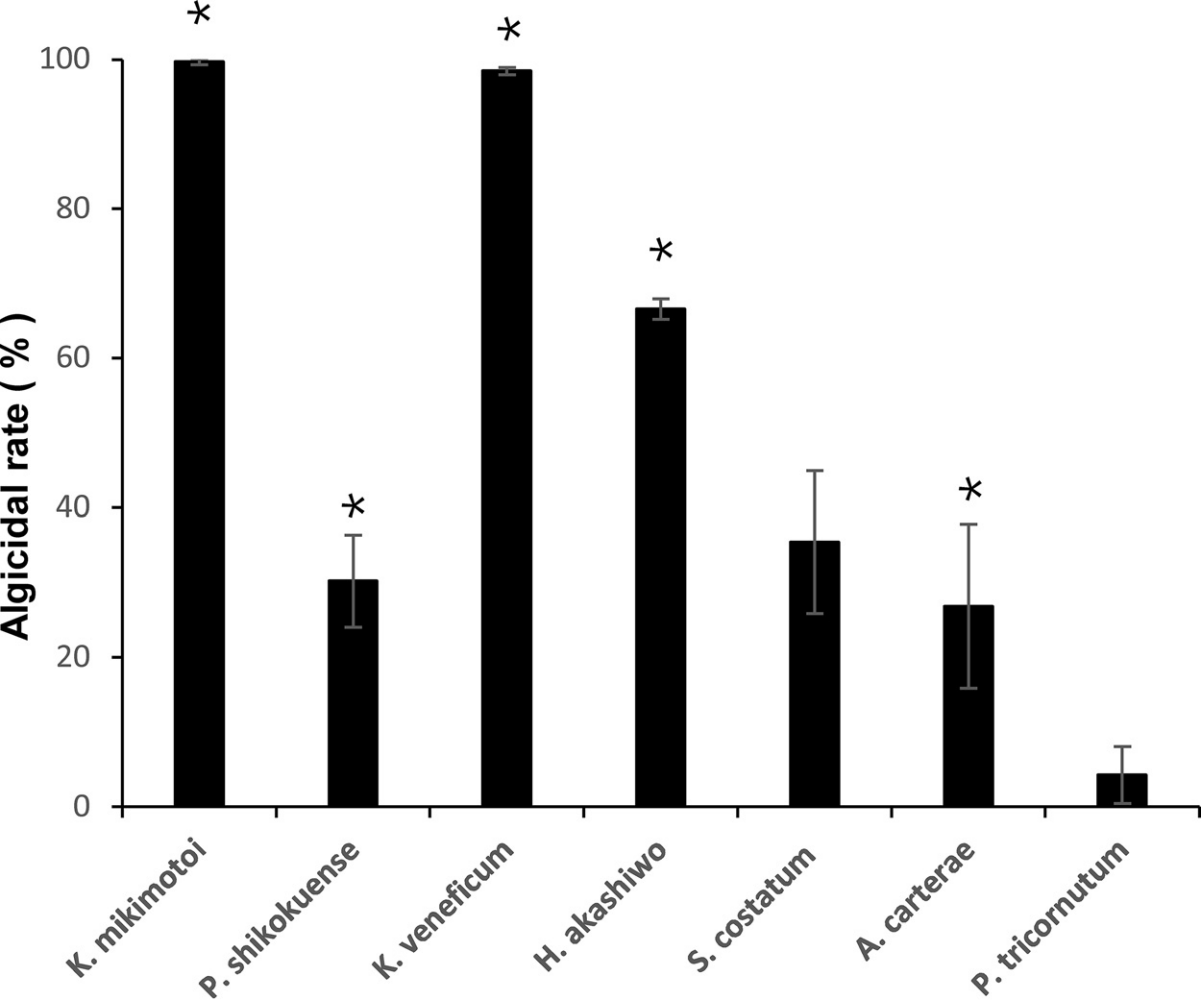
Algicidal activity of FDHY-MZ2 against several phytoplankters after 24 h of the challenge. Bars represent triplicate means of % activity. Error bars indicate ± standard deviation of biological triplicates. Statistical analyses were performed between cell density of FDHY-MZ2 treated culture and control culture. Black * represents statistical significance.

Strain FDHY-MZ2 had various negative effects on the growth of the tested phytoplankton (Fig. 5). At 24 h of the incubation period, FDHY-MZ2 showed the highest algicidal rate on *K. mikimotoi* (99.75% ± 0.42, *P* < 0.05) and K. veneficum (98.51% ± 0.49, *P* < 0.05). Significant negative effects were also observed on *H. akashiwo* (66.60% ± 1.39, *P* < 0.05), *P. shikokuense* (30.21% ± 6.16, *P* < 0.05) and A. carterae (26.82% ± 10.98, *P* < 0.05). However, FDHY-MZ2 had no statistical significance negative effect on the diatoms S. costatum (35.42% ± 9.55, *P* = 0.07) and *P. tricornutum* (4.23% ± 3.80, *P* = 0.63).

### Photochemical responses of *K. mikimotoi* to FDHY-MZ2 supernatant.

**FIG 6:**
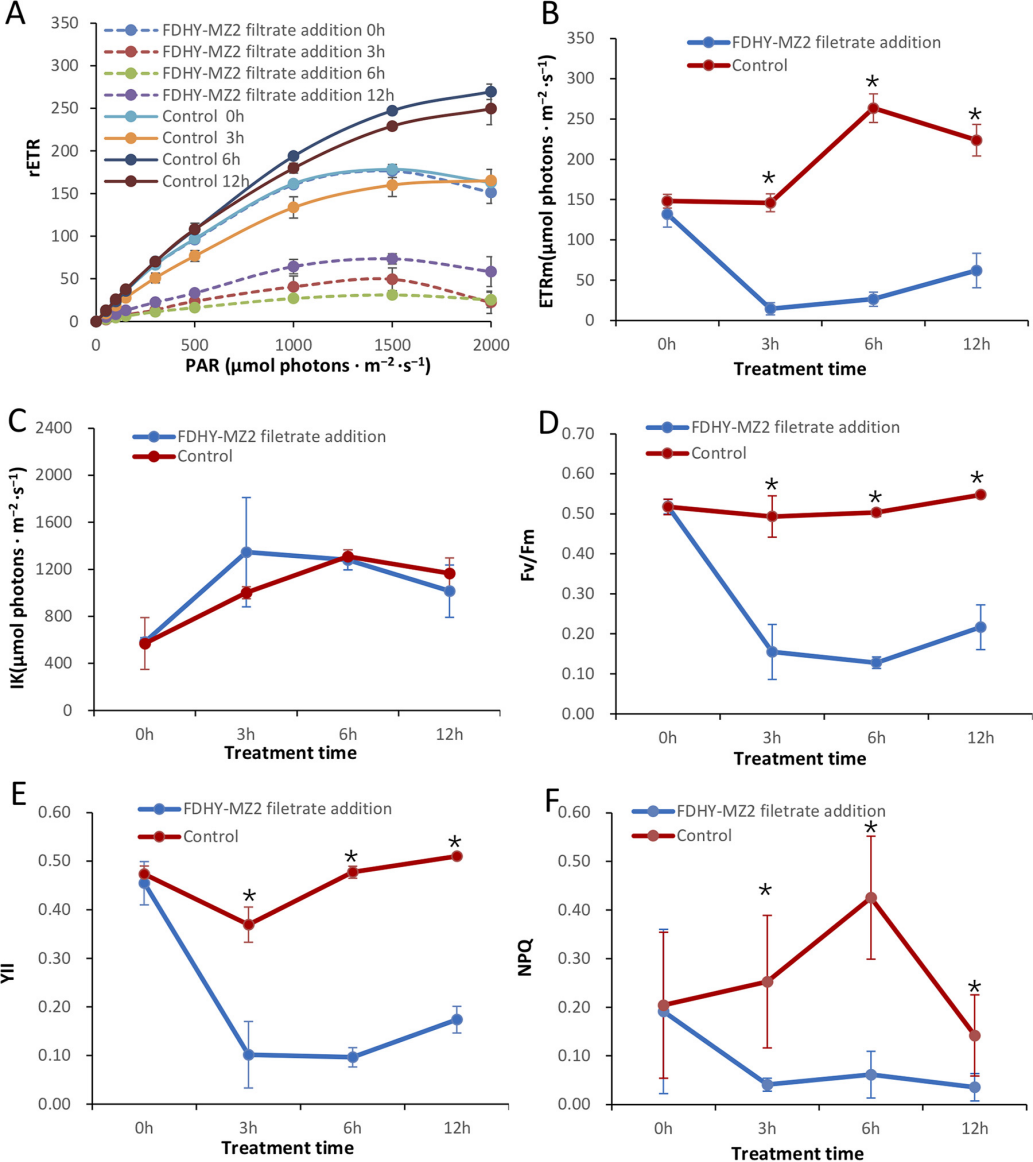
Photochemical traits of relative electron transport rate (rETR; A), maximal relative electron transport rate (rETRmax; B), saturating light intensity (I_k_; C), maximal photochemical quantum yield of PSII (Fv/Fm; D), effective quantum yield (YII; E) and nonphotochemical quenching (NPQ; F) in *K. mikimoto* with (blue line) or without (red line) supernatant treatment. Statistical analyses were performed between the two groups. Black * represents statistical significance.

To examine the effect of FDHY-MZ2 on the photosynthetic activity of *K. mikimotoi*, rETR, rETRmax, I_k_, Fv/Fm, YII and NPQ were measured in cultures of both groups (with and without FDHY-MZ2). All these parameters in *K. mikimotoi* were decreased significantly (*P* < 0.05) under FDHY-MZ2 supernatant treatment, except I_k_ (Fig. 6). With prolonged algicidal treatment, all influenced parameters obtained low values compared to the control group. The values of ETRm, Fv/Fm and YII showed increase with significant (*P* < 0.05) at 12 h in the algicidal-treated group.

### Regulation of functional genes in *K. mikimotoi* challenged by FDHY-MZ2.

**FIG 7:**
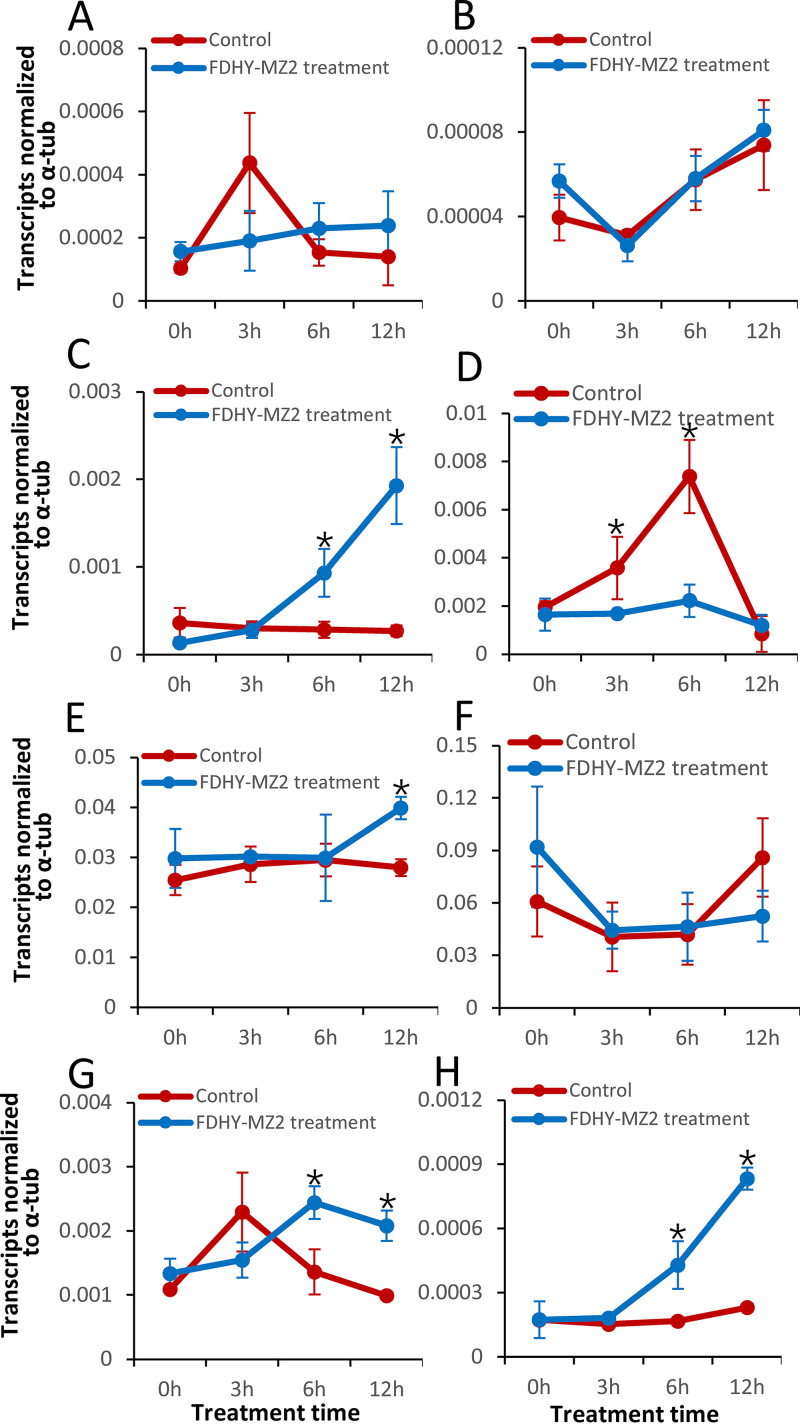
Gene transcription dynamics of functional genes normalized to reference gene α-tub with (blue line) or without (red line) supernatant treatment. (A) chlorophyll synthase (chlG), (B) glutamyl-tRNA reductase (hemA), (C) pheophorbide A oxygenase (PAO), (D) glutathione S–transferase (GST), (E) cyclin B, (F) proliferating cell nuclear antigen (PCNA), (G) cellulose synthase and (H) cellulase. Statistical analyses of gene expression level changes were performed between FDHY-MZ2 supernatant treated cell and control cell. Error bars indicate ± standard deviation of biological triplicates. Black * represents statistical significance.

In the RT-qPCR Experiment, the PCR efficiencies of target genes were all above 90% (Table S1). The expression levels of these genes were not detected after 24 h, as no cells were observed in the supernatant-treated group. Generally, these genes are involved in chloroplast synthesis and degradation chlorophyll synthase (chlG), glutamyl-tRNA reductase (hemA) and pheophorbide a oxygenase (PAO) (27), ROS scavenging (glutathione *S*-transferase) (28), cell cycle regulation (cyclin B and proliferating cell nuclear antigen, PCNA) (29, 30), and cell wall formation and deterioration (cellulose synthase and cellulase) (31). For chlG, hemA and PCNA genes, the expression profiles were not significantly changed (*P* > 0.05) with the addition of FDHY-MZ2 supernatant over the sampling period (Fig. 7A–B, F). For PAO, the transcripts were consistent at a relatively lower level in control cultures in the sampling period. The expression level of this gene in the treatment group gradually increased with significant (*P* < 0.05) after 3 h of exposure to the supernatant. Finally, the amplitude of PAO transcript was 3.32-and 7.11- fold higher in supernatant-treated cultures compared to the control group after 6 h and 12 h, respectively (Fig. 7C). Different expression patterns were also detected for glutathione *S*-transferase gene in the two groups. The transcript levels of this gene were consistently lower in supernatant-treated cultures during the sampling period. In the control cultures, the transcript abundance of this gene increased with significant (*P* < 0.05) and peaked at 6 h, with a 3.32-fold higher level in supernatant-treated cultures, followed by a decrease to the original level of sampling in the control group at time point 12 h (Fig. 7D). For cyclin B, the gene transcript abundance was consistent over the sampling period in control cultures. In FDHY-MZ2 supernatant-treated groups, the gene expression level increased with significant (*P* < 0.05) after 12 h treatment. The gene transcript abundance was significantly higher (*P* < 0.05) than that in control group at this time point (Fig. 7E). For gene cellulose synthase, the transcript abundance level showed a delayed pattern when cultures were treated with the supernatant (Fig. 7G). The expression profile of cellulase showed a pattern similar to that of PAO in treatment and control groups. In control cultures, the expression of cellulase was stable throughout the sampling period. In supernatant-treated cultures, transcript abundance showed levels similar to the control group within the first 3 h, followed by a steady amplitude increase with significant (*P* < 0.05) (Fig. 7H). At 6 and 12 h, the expression levels of this gene in the supernatant-treated cultures were 2.55 and 3.62 times higher than those in the control group.

### Bacterial agent powder production and application.

**FIG 8:**
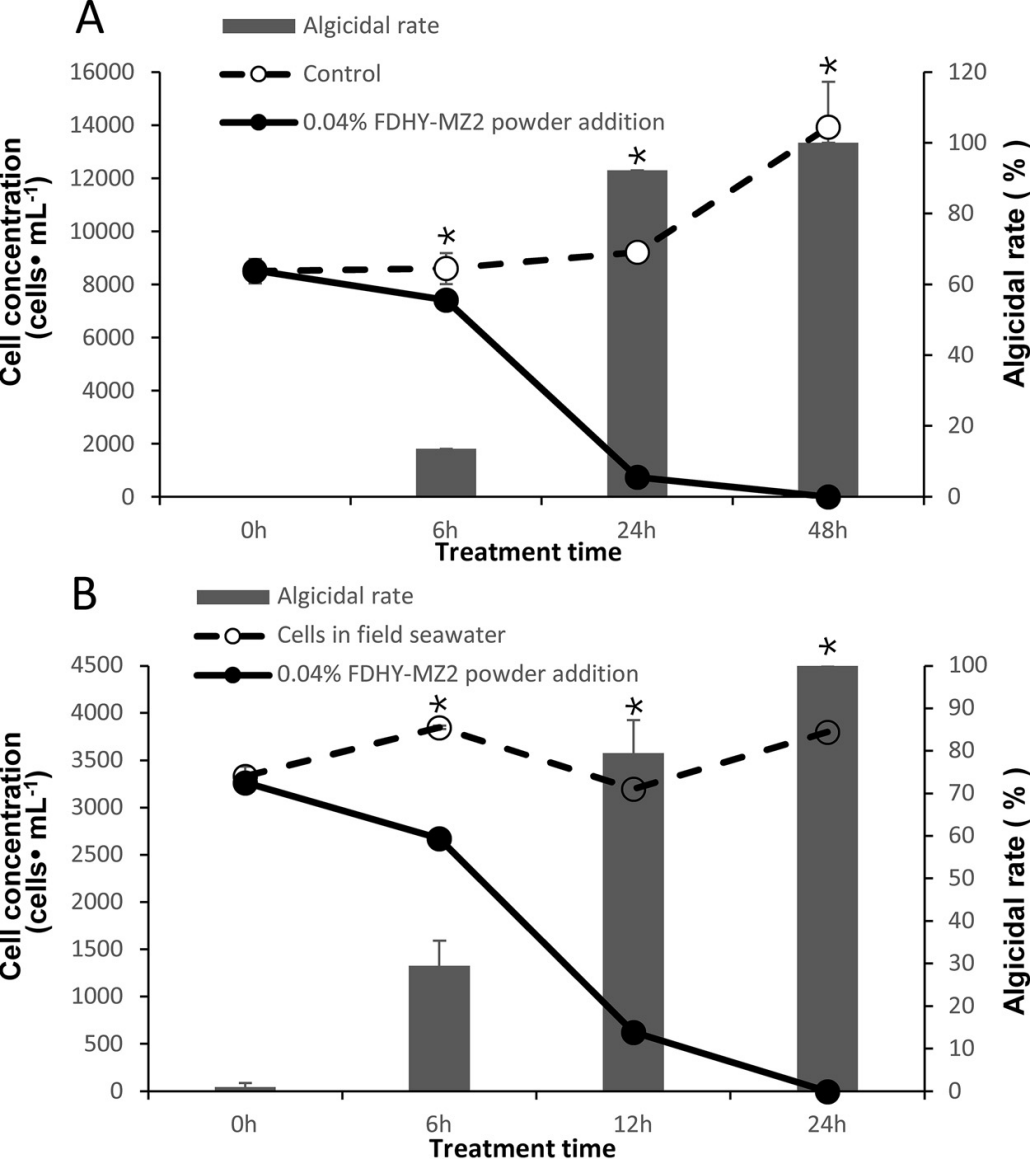
Algicidal activity of spray-dried powder produced with FDHY-MZ2 culture against a *K. mikimotoi* culture (A) and a field microcosm of *K. mikimotoi* bloom (B). The solid and the dashed lines indicate 0.04% (m/V) spray-dried powder-treated and control cultures, respectively. The gray bar indicates the algicidal rate of each sample. For A, statistical analyses were performed between cell density of FDHY-MZ2 spray-dried powder-treated culture and control powder treated culture. For B, statistical analyses were performed between cell density of FDHY-MZ2 spray-dried powder-treated culture and untreated culture. Error bars indicate ± standard deviation of biological triplicates. Black * represents statistical significance.

Using the bacterial culture grown at the stationary phase, the broth was sprayed using a laboratory spray drier, yielding algicidal power of 18.33 ± 0.24 g/L. To assess the algicidal activity of this powder, it was used to lyse laboratory *K. mikimotoi* cultures and naturally occurring *Karlodinium* sp.-dominated bloom. With the addition of 0.04% (m/V) algicidal powder, cultivated *K. mikimotoi* cells showed 92.20 and 100% decrease with significant (*P* < 0.05) after 24 and 48 h, respectively (Fig. 8). Using the same amount of algicidal powder, naturally blooming *Karlodinium* sp. cells were completely lysed after 24 h. The algicidal rate of 79.47% (statistical significance, *P* < 0.05) was observed after 12 h.

## DISCUSSION

Recent investigations have revealed that some bacterial lineage playing an important role in termination of HABs (5, 10). Thus, these microorganisms have been extensively studied. The dinoflagellate *K. mikimotoi* is a noxious algal bloom-forming species that causes aquaculture industry damaging widely in China costal area (22, 23). We expected to develop biological agents to inhibit the bloom formed by this species in costal aquaculture area. In this study, FDHY-MZ2, a bacterial strain isolated from the surface seawater of the East China Sea in a *K. mikimotoi* bloom event, exhibited strong algicidal activity against *K. mikimotoi*. Based on the RT-qPCR technique, the potential molecular response of *K. mikimotoi* to statin FDHY-MZ2 was investigated. To increase the performance of this strain and facilitate easy application, the broth was dried to obtain the algicidal powder, which was used on indoor *K. mikimotoi* cultures and a field microcosm of *Karlodinium* sp.-dominated bloom. The results of this study suggest that the developed bioagent is a potential source to inhibit *K. mikimotoi* blooms.

According to phylogenetic analysis, strain FDHY-MZ2 belongs to the genus *Pseudoalteromonas*, which contains some abundant algicidal bacteria in eutrophic marine environments (11) and can release algicidal components to lyse marine phytoplankton (32, 33). Several strains from this genus have algicidal activity against dinoflagellates (15, 34). These strains show different algicidal activities toward target microalgal cells. P. haloplanktis AFMB-008041 strain showed strong algicidal activity toward *Prorocentrum* species (32), whereas strain FDHY-MZ2, isolated in this study, showed a low algicidal rate toward *P. shikokuense*. In a similar study, with the addition of 10% *Pseudoalteromonas* sp. S-3, the algicidal rate on *K. mikimotoi* was 87% at 24 h (15). In this study, we observed a 100% algicidal rate after 24 h on *K. mikimotoi* when 0.5% FDHY-MZ2 was added to the *K. mikimotoi* culture. These results suggest that algicidal activity is species-specific. To develop a biological agent to control HABs, the algicidal rate is a central topic if the application in environmental engineering is envisioned. For such an approach, bacterial strains with high algicidal activities are required (7, 10). The algicidal rate of strain FDHY-MZ2 is higher than that of other reported algicidal bacteria against *K. mikimotoi* (15, 34, 35), making this strain an ideal source of bacterial agents to inhibit *K. mikimotoi* blooms.

Algicidal bacteria can damage the photosynthetic system of the target alga cell (16–19), which was also observed in this study, where significant photoinhibition in supernatant-treated algal cultures occurred, with marked declines in multiple photobiology parameters. The parameter NPQ was significantly lower in the treatments compared to the controls at time points 3 h, 6 h and 12 h. The decline of NPQ suggested that high light protection ability was lost (36). At the time points 3 h, 6 h and 12 h, the parameter rETR, Fv/Fm and YII was all decreased with significant in the treatments compared to the controls indicates the inhibition of the electron transport chain within the thylakoid membrane, resulting from a damaged photosynthetic system (36). The values of Fv/Fm and YII showed some recovery at time point 12 h in supernatant-treated groups compared to that was at time point 6 h. The recoveries were statistically significant. These recoveries may be related to the diel rhythm of photochemical parameter, which peaks at light- to dark-cycle transition or at the end of light cycle in dinoflagellate K. veneficum (37) and other phytoplankters (38, 39). In this study, the 12 h sampling time point was the light to dark transition, and the recovery photochemical parameters also existed in control groups at this time point. So, we speculated that this recovery might be caused by diel rhythm.

The electron flow disruption causes the generation of light-dependent H_2_O_2_. Elevated ROS accumulation is a common feature of algal cells exposed to algicidal bacteria (16). The production and scavenging of ROS are balanced in “normal” cells (40). The ROS scavenging activity related gene of the supernatant-challenged cells was detected using RT-qPCR for the glutathione *S*-transferase, which is an important ROS scavenging gene and protects the cell from oxidative damage (28). It is usually controlled by the circadian rhythms of antioxidants’ expression in dinoflagellate (41). The expression level of this gene was increased 3.32-fold in control cultures in the middle of the sampling period compared with a stable expression profile in the supernatant-treated cultures. These results suggest that the ROS scavenging system related gene was inhibited by algicidal bacteria, which explains the ROS accumulation in phytoplankton subjected to algicidal bacteria.

ROS are linked to programmed cell death (PCD) (40, 42). While cellulase is involved in the degradation of cell walls (43), algicidal bacteria can destroy the walls of target algal cells (8, 9) via the excretion of extracellular algicidal compounds. However, the responses to cell wall degradation and modification by algal cells are largely unknown. We selected gene cellulose synthase and cellulase, which have a role in cell wall formation and degradation, to detect the molecular responses of the cell wall induced by algicidal bacteria. In higher plants, cellulase is involved in the degradation of cell walls in PCD (43), and its activity is generally coupled with the autolysis of plant cells (44). Furthermore, PCD is a process in several dinoflagellates caused by algicidal bacteria. The increased gene expression level of cellulase was coupled with cell death in *K. mikimotoi*. We deduced that FDHY-MZ2 induced algal cell wall degradation through increased cellulase activity, which is linked to PCD. This process can be confirmed by examining the caspase-like activity (45). With the increased expression level of cellulase, the transcript abundance of cellulose synthase showed a delayed expression dynamically. The expression level of this gene commonly can adapt to cell growth to environmental stresses (46). The cellulose synthase in the later part of the sampling period may be upregulated to form cyst under biotic stress (47, 48). However, the possibility of a response needs further investigation.

When challenged by algicidal bacteria, the target algae cell responds by the degradation of photosynthetic pigments (17). In this study, genes related to chloroplast synthesis and degradation were selected to determine the molecular responses of algal cells to algicidal bacteria. PAO, the key gene in the breakdown of chlorophyll via disruption of the light-harvesting complex (49, 50), was largely induced by algicidal supernatant, suggesting increased chloroplast degradation, which results is consistent with reports in which the cellular photosynthetic pigments of algae are decreased by algicidal bacteria (17, 51). The active site of gene PAO is located in the envelope of chloroplast (52). It is induced by various abiotic and biotic stresses and plays an important role in the defense system (49, 53, 54). For the *K. mikimotoi* culture, algicidal bacteria represent a type of biotic stress, and survival cells may induce PAO expression as a response mechanism. However, the chlG and hemA genes were not affected by algicidal bacteria over the sampling period. The chlG and hemA are two important genes in chlorophyll biosynthesis (55). Based on statistical analysis, the transcript levels of chlG and hemA genes were not changed significantly with algicidal supernatant treatment. A previous report indicated that chlG and hemA acted on thylakoild (52), which are located in the central part of chloroplast and packed by three layers of plastids membranes in dinoflagellate (56). The expression of them was controlled by feedback, and their transcript abundance was downstream metabolic flow related (57). We speculated that the no influenced expression profile of the two genes in the sampling period might relate to good protection of thylakoild by outer plastid membranes in surviving cells.

Algicides inhibit cell cycle progression and can sometimes induce programmed cell death because of DNA degradation (21, 58). To cope with DNA damage, the cells activate cell cycle checkpoints and rapidly reach the S phase to repair the damaged nucleic acids (21). Cyclin B is an important checkpoint gene in dinoflagellates, and its expression rises from the S phase until the G2M phase (29). This expression profile has been detected in human cells (59). In our study, the transcript abundance of cyclin B was elevated after 12 h of treatment with algicidal supernatant, suggesting that the S phase was put forward. This response of the algal cells may launch repair DNA mechanisms.

We used the spray-drying method to enrich the algicidal compounds of the FDHY-MZ2 culture. This method is widely used to preserve bacteria (60), but data regarding algicidal bacteria are still scarce. In this study, we tested spray-dried powder against a laboratory *K. mikimotoi* culture and a field mesocosm of *Karlodinium* sp. bloom. The algicidal rates were 92.2 and 100%, respectively, over 24 h when 0.04% (m/v) mass fraction powder was added. Both the genera *Karenia* and *Karlodinium* belong to the family Kareniaceae, with similar cell structures and ecological characteristics. They are unarmored dinoflagellates and contain endosymbionts of haptophyte origin, which function as photosynthetic plastids. FDHY-MZ2 shows high algicidal activity on Kareniaceae, at least on some members of it. Our study provides a new strategy for the control of harmful algal bloom-forming species *in situ*. Meanwhile, we also realized that this is a preliminary experiment to evaluate the algicidal powder as a means to control *Karlodinium* blooms in the field. More data may need to be collected to support this in the future, including the impacts on nontarget species.

### Conclusions.

*Pseudoalteromonas* sp. strain FDHY-MZ2 is a novel marine bacterial strain with strong algicidal activity against *K. mikimotoi*. It secretes extracellular algicidal compounds that completely lyse *K. mikimotoi* cells within 24 h. During algal cell lysis, the algal photosynthetic system was damaged. The FDHY-MZ2 supernatant induced the expression of cellulase, PAO and cyclin B genes, suggesting enhanced cell wall deterioration, chloroplast degradation and cell cycle regulation of *K. mikimotoi*. The ROS scavenging genes were significantly downregulated, indicating that the ROS removal system was damaged. We found that the expression levels of chlG and hemA genes were not influenced. A delayed expression pattern of the cellulose synthase gene during the sampling period was also observed. Because of the high algicidal activity of strain FDHY-MZ2, this strain was used to produce an algicidal powder, showing high algicidal activity against indoor algal culture and microcosm of nature algal bloom. These findings show that strain FDHY-MZ2 can inhibit harmful algal blooms, especially for *K. mikimotoi* and *Karlodinium* sp. dominated bloom events. For viable use of this strain in real events, biosafety and biosecurity risks evaluation should be investigated in the future.

## METHODS

### *K. mikimotoi* cultures.

The *K. mikimotoi* culture was obtained from the Center for Collections of Marine Algae at Xiamen University, China (CCM-083 strain). The culture was grown in a glass bottle with L1-Si medium (61) prepared with filtered (0.22 mm), autoclaved seawater (28 PSU). To minimize the influence of contaminated bacteria, the culture was treated with antibiotics (final concentrations of ampicillin, kanamycin and streptomycin were 200 mg/L, 100 mg/L and 100 mg/L, respectively) as reported previously (62) and maintained at 20 ± 1°C under illumination at a light intensity of 100 μmol photons·m^−2^·s^−1^ and a 14:10 h light: dark cycle. To monitor the growth rate of the culture, 1 mL of sample was fixed with Lugol's, and cell numbers were counted using a Sedgwick-Rafter counting chamber under a fluorescence microscope (Nikon ECLIPSE 80i, Tokyo, Japan). Exponential-phase algal cultures were prepared for algal lysis experiments under laboratory conditions.

### Screening for alga-lysing bacteria and isolation of strain FDHY-MZ2.

A surface seawater sample was collected from the Lianjiang sea area of China, in which a *K. mikimotoi* bloom occurred on June 9, 2018. The sample was prefiltered through a 100-μm-pore size Nitex^R^ filter (Huading filter Corp., Hangzhou, China), and the filtrate was serially diluted (10-fold) using sterile seawater 5 times; and 0.1-mL aliquots of each dilution were spread onto Zobell 2216E agar plates (Dalong Corp., Taizhou, China), followed by incubation at 25°C for 7 days. Individual colonies were picked and separately inoculated into liquid 2216E broth at 25°C for 24 h at 150 rpm. For each individual bacterial culture, 0.5 mL was added to triplicate 25-mL aliquots of exponentially growing *K. mikimotoi* cultures. Subsequently, the cultures were incubated under the condition that algal culture prepared as described above. For a negative control, the same volume of sterile 2216E medium was added into the algal cultures. The cell density was monitored using a Sedgwick-Rafter counting chamber under microscope and the algicidal rate calculated using the following equation:

Algicidal rate (%) = (NC – NT)/NC × 100

Where NT represents the algal cell density in the treated algal culture; NC is the algal cell density in the negative control. For each counting assay, the experiment was done in 15 min. All experiments were performed in triplicate. A bacterial strain that showed an algicidal rate over 80% within 48 h was defined as algicidal (9). Among the isolated algicidal bacteria, FDHY-MZ2 showed the highest algicidal activity against K. mikimotoi. Thus, we selected this strain for further study.

### Identification and algicidal activity of strain FDHY-MZ2.

Strain FDHY-MZ2 was characterized using 16S rRNA gene analysis. The genomic DNA (gDNA) was extracted using the CTAB method combined with the Zymo DNA Clean & Concentrator kit (Zymo Research Corp., Orange, CA, USA) as reported previously (9). The 16S rRNA gene was amplified by PCR using 27F and 1492R primers (63). The obtained PCR product was purified and cloned using Zymo gel DNA recovery kit (Zymo Research Corp., Orange, CA) and TAKRA clone kit (TaKaRa Biotechnology, Dalian, China), respectively, according to the manufacturer’s instructions. Then the clone was sent to BGI (Beijing Genomics Institute, Beijing, China) for sequencing. Phylogenetic analysis was performed to determine the association of the strain FDHY-MZ2 with previously characterized bacteria based on their 16S rDNA sequences. The National Center for Biotechnology Information (NCBI)’s nonredundant (nr) database was used to obtain reference sequences by Basic Local Alignment Search Tool (BLAST) search (64), which were aligned using ClustalX (65). The phylogenetic tree was constructed using the neighbor-joining method in the MEGA software package (v5.0) (66), with 1,000 resamplings in bootstrapping.

### Algicidal mode and lytic effects on various HAB species of strain FDHY-MZ2.

To investigate the algicidal activity of the bacterial strain FDHY-MZ2 against *K. mikimotoi*, 0.2, 0.5 and 1% volumes (vol/vol) of the stationary-phased bacterial strain were inoculated into 20 mL *K. mikimotoi* cultures. The same serial volumes of 2216E broth were added to algal cultures as controls. Algal cell density and algicidal rate were measured using the formula provided above.

To determine the algicidal mode of action of strain FDHY-MZ2, cell-free supernatant and bacterial cells from the strain were collected by filtration and centrifugation, respectively, and separately incubated with *K. mikimotoi* cultures. For each fragment collection, 20 mL stationary-phased bacterial culture was prepared. Cell-free supernatant was collected using a 0.22-μm polycarbonate membrane filter (Merck Millipore), and the bacterial cells were collected by centrifugation at 1,770 G for 5 min at room temperature. The bacterial cell pellets were resuspended using the 20 mL of sterile L1 medium, and the supernatant and the resuspended cell pellets were added into algal cultures at a final volume rate of 1% (vol/vol). On the incubation time point 6 h, 12 h, 18 h and 24 h, the algicidal rate was calculated as described above.

The algal-lytic activity of strain FDHY-MZ2 was also detected for other algal species, including dinoflagellate species *Prorocentrum shikokuense*, K. veneficum and Amphidinium carterae, diatom species S. costatum and *Phaeodactylum tricornutum*, Raphidophyceae species Heterosigma akashiwo. These cultures were cultured in the same medium and under the same temperature and light conditions as described above for *K. mikimotoi*. For each algal species, FDHY-MZ2 was added to algal cultures at a final ratio of 1.0% (vol/vol). Algal cell density was monitored for 24 h after the addition of the bacteria, and the algicidal rate was calculated as described above. The statistical analysis was performed between treatment and control groups for their survival cell density.

### Algicidal process observation of *K. mikimotoi* attacked by FDHY-MZ2.

To determine the algicidal process of *K. mikimotoi*, a series of exponentially growing *K. mikimotoi* cultures were prepared for microscopic examination. Briefly, 200 μL of the bacterial supernatant was added to 20 mL algal cultures for each time point, and the incubated algal cells were observed under a microscope at 0, 6, 12 and 24 h. Meanwhile, 10 mL of incubated algal culture at each time point was collected by centrifugation at 1,770 G for 5 min at 20°C, and the cell pellet was resuspended in 0.1 M sodium phosphate buffer solution (PBS, pH 7.4, 80 g NaCl, 2.0 g KCl, 21.7 g Na_2_HPO_4_ · 7H_2_O, 2.59 g KH_2_PO_4_, prepare in 1 L of ddH_2_O.) with 4% (vol/vol) paraformaldehyde for 10 min. The cells were then rinsed twice in PBS and stained for 5 min in the dark at room temperature with 5 g/mL 4′,6-diamidino-2-phenylindole (DAPI). Fluorescence microscopy (Nikon ECLIPSE 80i, Tokyo, Japan) was used to examine the bright-field and fluorescence of the samples.

### Photochemical responses of *K. mikimotoi* with FDHY-MZ2 challenging.

To examine the photochemical responses of the algal cells to FDHY-MZ2, photochemical traits, such as relative electron transport rate (rETR), maximal relative electron transport rate (rETRmax), saturating light intensity (Ik), maximal photochemical quantum yield of PSII (Fv/Fm), effective quantum yield (YII) and nonphotochemical quenching (NPQ) were measured in cultures of both control and FDHY-MZ2 supernatant treated groups using a pulse-modulated fluorimeter (PAM-2100, Waltz, Effeltrich, Germany). In the study, the control group was grown in the L1 medium as described above. The algicidal treatment group was grown in the L1 medium with the addition of 1% (vol/vol) FDHY-MZ2 strain culture supernatant. At 0, 3, 6, 12 and 24 h, the samples were collected for photochemical parameter measurement. NPQ was calculated by the equation: NPQ=(Fm-Fm’)/Fm’ (36), where Fm represents the maximum chlorophyll fluorescence yield in the dark-adapted states and Fm’ is the maximum chlorophyll fluorescence yield at actinic light levels. The RLC is the relative electron transport rate in response to nine different and increasing actinic light intensities (0, 50, 100, 150, 300, 500, 1000, 1500 and 2000 μmol photons·m^−2^·s^−1^) with a 10 s for each increment separated by a 0.8 s saturating pulse (5000 μmol photons·m^−2^·s^−1^). The equations were calculated for the rest photochemical parameters in accordance with a previously published report (52).

### Gene expression analysis using reverse transcription quantitative PCR (RT-qPCR).

To illustrate the molecular response mechanism of *K. mikimotoi* to FDHY-MZ2, the expression profiles of genes related to chloroplast synthesis and degradation, cell wall formation and deterioration and cell cycle regulation of *K. mikimotoi* were observed. The samples were collected with a photochemical parameter measurement sample. At 0, 3, 6, 12 and 24 h, 200 mL samples were collected by centrifugation at 7,080 G for 5 min at 4°C, and the obtained cell pellets were resuspended in 1 mL TRIzol Reagent (Invitrogen, Waltham, MA, USA) for total RNA extraction.

Total RNA was extracted using the TRI-Reagent method coupled with the Direct-zol RNA Miniprep kit (Zymo Research, Irvine, CA, USA) as reported previously (67). Potentially contaminating gDNA was removed using RQ1 DNase (Promega, Madison, WI, USA) according to the manufacturer’s instructions. The expression levels of the genes of interest in the different samples were determined using RT-qPCR. M-MLV reverse transcriptase (Promega, Madison, WI, USA) was used to synthesize complementary DNA (cDNA) from 1 μg of total RNA, which was also used for specific gene isolation. Briefly, 100 ng of oligo-(dT)16 primer was used to prime cDNA synthesis for each sample. The target gene cDNA fragment was amplified using primers designed for qPCR (Table S1). The primers were designed according to a previously reported *K. mikimotoi* transcriptome (68), and the sequences of these genes were submitted to NCBI (cellulase, cellulose synthase, chlorophyll synthase, cyclin B, glutathione *S*-transferase, proliferating cell nuclear antigen, pheophorbide a oxygenase and glutamyl-tRNA reductase; GenBank accession number OM101026-OM101033). The fragment was cloned and amplified using PCR to prepare a standard curve as previously described (67). All qPCRs were performed on a CFX96 real-time PCR system (Bio-Rad, Hercules, CA, USA) with iQTM SYBR Green Supermix (Bio-Rad, Hercules, CA, USA). Each reaction was carried out in a total volume of 12 μL containing 250 nM each primer, 5 μL cDNA, and 6 μL SYBRH Green Supermix. For both the 10-fold dilution series of the standard and the experimental cDNA, qPCR was done to obtain technical replicates for each of the biological triplicates. Reference gene α-tub (69) was included in the qPCR as a reference gene for the normalization of target gene expression levels. The abundances of the target gene and reference gene transcripts were analyzed using the CFX software (Bio-Rad, Hercules, CA, USA).

### Preparation of bacterial agent powder and its application.

To produce easy-to-use ingredients to lyse harmful algae, the fermentation broth (prepared as mentioned above) was sprayed using a lab spray drier (Naai Precision Instrument Co., Ltd. Inc. Shanghai, China; temperature of 140°C; flow rate of 350 mL · h^−1^). A cyclone separator separated the powder from the hot gases and collected it in the collecting vessel of the separator. The bacterial agent powder obtained over a 4 h drying period for fermentation broth and the resultant powder was collected. The control powder was also prepared with the condition that was described for bacterial agent powder, which was no FDHY-MZ2 bacterium innovated in original medium broth. The powder was used to lyse the laboratory culture and naturally occurring *Karlodinium* sp. dominated bloom (May 24–29, 2019. Pingtan, China) (26). For both laboratory culture and naturally bloom seawater, the bacterial agent powder was added at a final mass fraction of 0.04% (m/V). This algal lysis test was performed in a 1 L glass bottle for *K. mikimotoi* laboratory culture and a 30 L tank for field bloomed *Karlodinium* sp. Algal cell density and algicidal rate were measured using the above formula.

### Statistical analyses.

To examine the significant differences of algal growth, gene expression and photobiology parameters between treatments and controls at each of the time points, one-way ANOVA statistical test was performed using SPSS 19.0 software package, and *P* < 0.05 was considered to indicate significance. To detect the significant differences of photobiology parameters between FDHY-MZ2 treated culture at time point h 12 and previous time point, the same one-way ANOVA statistical test as described above was performed for statistical analyses.
