# Supplementary material for: A Novel Algicidal Bacterium and Its Effects against the Toxic Dinoflagellate Karenia mikimotoi (Dinophyceae)
Source: Microbiol Spectr. 2022 May 26;10(3):e00429-22. doi: 10.1128/spectrum.00429-22 (PMC9241683; doi:10.1128/spectrum.00429-22)
Supplement: SUPPLEMENTAL FILE 1 — Supplemental material. Download spectrum.00429-22-s001.pdf, PDF file, 0.4 MB [file spectrum.00429-22-s001.pdf]

Table S1 Primers used in this study.

Primer name	Sequences (5'–3')	Application	PCR efficiency	Source
Km_α-tub_qF	AGCCACCTACCGTTGTGCCTG	K. mikimotoi α-tub qPCR (forward)	91.7%	(1)
Km_α-tub_qR	TGGTCGATGCGCGAGAAGAC	K. mikimotoi α-tub qPCR (reverse)		
Km_GST_qF	TGCACCCATTCTCAGCTCTGA	K. mikimotoi glutathione S-transferase qPCR (forward)	90.2%	This study
Km_GST_qR	TCATGCGCGATGTTCCACTC	K. mikimotoi glutathione S-transferase qPCR (reverse)		This study
Km_PCNA_qF	GTGGCAGAATGATGCAGAC	K. mikimotoi PCNA qPCR (forward)	90.3%	This study
Km_PCNA_qR	AAGCGAATACCCTCCTTGC	K. mikimotoi PCNA qPCR (reverse)		This study
Km_ cyclinB_qF	GAGGTAGCCATGCTCACAGA	K. mikimotoi cyclin B qPCR (forward)	92.7%	This study
Km_cyclinB_qR	TGATGGACTGTATCGGATCA	K. mikimotoi cyclin B qPCR (reverse)		This study
Km_CS_qF	TGGATCACAGTTGCGTCCA	K. mikimotoi ChlG qPCR (forward)	95.6%	This study
Km_CS_qR	GCCTGGTACTTCACATCGTTA	K. mikimotoi ChlG qPCR (reverse)		This study
Km_hemA_qF	CAGTTGACCCTATCATTAC	K. mikimotoi hemA qPCR (forward)	93.2%	This study
Km_hemA_qR	ATGCTCGCTTGGCCTGATTG	K. mikimotoi hemA qPCR (reverse)		This study
Km_PAO_qF	CCAGGAAGCTGCGCAGATA	K. mikimotoi Pheophorbide a oxygenase qPCR (forward)	91.1%	This study
Km_PAO_qR	TTGCCAAGTGTGCCATGGTGT	K. mikimotoi Pheophorbide a oxygenase qPCR (reverse)		This study
Km_cellulose stha_qF	GATTACAGGCAGGCAGCCA	K. mikimotoi cellulose synthase qPCR (forward)	94.7%	This study
Km_cellulose stha_qR	ACAGCAGGATTAGGACCCCA	K. mikimotoi cellulose synthase qPCR (reverse)		This study
Km_cellulase_qF	CATATCATGAGGACACTGTGC	K. mikimotoi cellulase qPCR (forward)	92.8%	This study
Km_cellulase_qR	GAGATGTTCAAGATGCTCAACA	K. mikimotoi cellulase qPCR (reverse)		This study

REFERENCES

1. Shi X, Xiao Y, Liu L, Xie Y, Ma R, Chen J. 2020. Exploring reliable reference genes for gene expression normalization in Karenia mikimotoi using real-time PCR. Journal of Applied Phycology 32:431–440.
